# Breaking dogmas: the plant vascular pathogen *Xanthomonas albilineans* is able to invade non-vascular tissues despite its reduced genome

**DOI:** 10.1098/rsob.130116

**Published:** 2014-02-12

**Authors:** Imène Mensi, Marie-Stéphanie Vernerey, Daniel Gargani, Michel Nicole, Philippe Rott

**Affiliations:** 1 CIRAD, UMR BGPI, TA A-54/K, Montpellier Cedex 5 34398, France; 2 IRD, UMR RPB, BP 64501, Montpellier Cedex 5 34394, France

**Keywords:** *Xanthomonas albilineans*, confocal laser scanning microscopy, cytochemistry, transmission electron microscopy, non-vascular plant tissue

## Abstract

*Xanthomonas albilineans*, the causal agent of sugarcane leaf scald, is missing the Hrp type III secretion system that is used by many Gram-negative bacteria to colonize their host. Until now, this pathogen was considered as strictly limited to the xylem of sugarcane. We used confocal laser scanning microscopy, immunocytochemistry and transmission electron microscopy (TEM) to investigate the localization of *X. albilineans* in diseased sugarcane. Sugarcane plants were inoculated with strains of the pathogen labelled with a green fluorescent protein. Confocal microscopy observations of symptomatic leaves confirmed the presence of the pathogen in the protoxylem and metaxylem; however, *X. albilineans* was also observed in phloem, parenchyma and bulliform cells of the infected leaves. Similarly, vascular bundles of infected sugarcane stalks were invaded by *X. albilineans*. Surprisingly, the pathogen was also observed in apparently intact storage cells of the stalk and in intercellular spaces between these cells. Most of these observations made by confocal microscopy were confirmed by TEM. The pathogen exits the xylem following cell wall and middle lamellae degradation, thus creating openings to reach parenchyma cells. This is the first description of a plant pathogenic vascular bacterium invading apparently intact non-vascular plant tissues and multiplying in parenchyma cells.

## 2. Introduction

Plants are an important source of water and nutrients for microbes, and they are the hosts of numerous bacterial pathogens from both Proteobacteria and Actinobacteria phyla. During their parasitic lifestyle, plant pathogenic bacteria show a high degree of tissue specificity and they invade either the intercellular spaces of the mesophyll tissue (apoplastic pathogens) or the vascular system (vascular pathogens) of the host [1,2]. The mesophyll tissue is located between the upper and lower layers of leaf epidermis, and both mesophyll tissue and leaf epidermis contain parenchyma cells. The plant vascular system is composed of xylem elements (dead vessels with lignified cell walls), which ensure the transport of water and nutrients from the roots to the leaves, and phloem elements (tissue with living cells), which control the transport of photosynthetic products from mature leaves to other organs (notably storage organs). Although phloem is nutrient-rich, most bacterial vascular pathogens multiply in the xylem, a poor-nutrient environment [3]. Furthermore, at the scale of microbes, these tissues represent a real battlefield in which pathogenic and non-pathogenic bacteria are subjected to several and various layers of defence.

A major sophisticated strategy employed by successful bacterial pathogens is to assault key intracellular host processes to exploit the attractive nutritional menu provided by their eukaryotic hosts and to evade or suppress host defences. To achieve this goal, numerous Gram-negative plant and animal pathogenic bacteria rely on a specialized type III secretion system (T3SS) to inject virulence proteins or effectors directly into the cytosol of host cells and thus allow manipulation of a variety of host cellular processes to their own benefit [4–6].

In contrast to most Gram-negative pathogenic bacteria, *Xanthomonas albilineans*, the causal agent of sugarcane leaf scald, does not possesses a hypersensitive response and pathogenicity (Hrp) T3SS [7]. Additionally, this particular species of the *Xanthomonas* genus has a reduced genome (3.8 Mb) in comparison with the other sequenced plant pathogenic xanthomonads [7,8]. Bacteria with reduced genomes have often been associated with a high degree of niche specialization and restriction to specific tissues in their hosts [9–12]. Similar to *X. albilineans*, *Leifsonia xyli* subsp. *xyli* (the causal agent of ratoon stunting disease of sugarcane), *Xylella fastidiosa* (the causal agent of a lethal disease in numerous hosts) and *Ralstonia syzygii* (the causal agent of Sumatra disease of cloves) have experienced a reduction in genome size during their speciation (2.6, 2.7 and 3.6 Mb, respectively) [13–15]. These latter three pathogens are known as fastidious xylem-limited bacteria that live exclusively in xylem vessels or tracheary elements [11,16–18].

The genome of *Candidatus* Liberibacter asiaticus (causal agent of citrus huanglongbing) is typical of drastically reduced genomes (1.2 Mb, the smallest genome of all sequenced pathogenic bacteria) [19,20], and this pathogen is strictly adapted to sieve cells of phloem vessels [21,22] and salivary glands of citrus psyllids [23], natural vectors of the pathogen. Furthermore, phytoplasmas, which are wall-less plant pathogenic bacteria from the class of Mollicutes, are limited to the nutrient-rich phloem [24] and are characterized by genome downsizing (0.5–1.3 Mb) [25].

*Xanthomonas albilineans* generates various leaf and stalk symptoms during the disease progress [26,27]. This pathogen causes the appearance of white, narrow, sharply defined leaf stripes, leading to complete wilting and necrosis of infected leaves, and to plant death. Until now, *X. albilineans* was considered as a xylem-limited pathogen based on cytological observations of the pathogen in infected leaf tissues [28,29]. Even if leaf scald symptoms result from changes in the chlorenchyma cells, *X. albilineans* was considered as constrained within the xylem [29]. However, other observations suggested that, in contrast to *L. xyli* subsp. *xyli, X. albilineans* is not limited to the xylem but is also present in surrounding tissues. In 1932, Martin *et al*. [30] reported that, in the stalk of susceptible plants, the pathogen spreads within the xylem, dissolves the cells surrounding the annular vessels of the bundles and thus leads to the formation of lysigenic cavities. Additionally, when imprints of sugarcane stalks infected by *X. albilineans* are made on selective medium, growth of the pathogen is observed on the entire imprint area and not only in the vascular areas (see electronic supplementary material, figure S1). Similarly, when infected stalks are used for stalk blot immunoassays, the entire section shows a positive reaction, in contrast to stalks infected by *L. xyli* subsp. *xyli* that show only a positive reaction in the vascular bundle imprints [31].

Recently, observations of semi-thin sections of infected leaves treated with *X. albilineans* anti-serum suggested that the pathogen is not restricted to the xylem but is also present in the epidermal cuticle, epidermal cell (EC) walls, guard cells, subepidermal fibres and pericyclic fibres [32]. In order to elucidate the accurate location of the leaf scald pathogen *in planta*, we used herein three different techniques to visualize the bacteria in infected sugarcane tissues: (i) confocal microscopy with fresh material, (ii) confocal microscopy with fixed samples treated with *X. albilineans* anti-serum and (iii) transmission electron microscopy (TEM). We demonstrate that *X. albilineans* is not only able to infect and to move within the xylem, but is also able to invade apparently intact parenchymatous cells, as well as other non-vascular cells, a feature that has not been described for any plant pathogenic bacterium so far.

## 3. Material and methods

### 3.1. Bacterial strains and culture conditions

Characteristics of bacterial strains and plasmids used in this study are presented in table 1. Bacteria were cultured routinely at 28°C on modified Wilbrink's (MW) agar medium containing sucrose (10 g), peptone (5 g), K_2_HPO_4_-3H_2_O (0.50 g), MgSO_4_-7H_2_O (0.25 g), Na_2_SO_3_ (0.05 g), agar (15 g), distilled water (1 l), pH 6.8–7.0 [38]. Strains deriving from wild-type strains XaFL07-1 (from Florida) and GPE PC73 (from Guadeloupe) were produced and cultured on the same medium supplemented with appropriate antibiotics, as described below. All strains and mutants of *X. albilineans* were stored at −80°C as turbid water suspensions and retrieved before the preparation of electrocompetent cells or plant inoculation by plating on MW agar medium supplemented with appropriate antibiotics. *Escherichia coli* strains were grown on Luria-Bertani (LB) agar or in LB broth at 37°C and were used and stored according to standard protocols [39]. Antibiotics were used at the following concentrations: ampicillin 50 µg ml^−1^, gentamicin 3 µg ml^−1^ and kanamycin 20 µg ml^−1^, for *E. coli* strains and *X. albilineans* strains.

**Table 1.** Characteristics of plasmids and bacterial strains used in this study. Amp, ampicillin; Gm, gentamycin; Kn, kanamycin; Rif, rifampicin.

strain or plasmid	relevant characteristics	reference
plasmids		
pUFZ75	P_trp_-TIR-*gfp* cassette in pUFR034, Kn^r^	[33]
pUFR047	IncW, Mob+, lacZα+, Par+, Gm^r^, Amp^r^	[34]
pUFR047-GFP	*Kpn*I/*Sal*I fragment from pUFZ75 cloned into pUFR047, Gm^r^, Amp^r^	this work
*E. coli* strains		
*E. coli* DH5α	F-, *endA1*, *hsdR17*(rk-,mk+), *supE44*, *thi-1*, *recA1, gyrA96*, *relA1*, *Φ*80*lacZ*ΔM15, Δ(*lacZYA-argF*)*U169*	Gibco-BRL
*X. albilineans* strains		
GPE PC73	wild-type strain from Guadeloupe	[7]
XaFL07–1	wild-type strain from Florida	[35]
*X. albilineans* derivatives		
M427	*xanB*::Tn*5* derivative of strain XaFL07-1, defective in biosynthesis of surface polysaccharides, Kn^r^	[35]
M903	XALc_2705::Tn*5* derivative of strain XaFL07-1, defective in biosynthesis of surface polysaccharides, Kn^r^	[35]
M967	*gmd*::Tn*5* derivative of strain XaFL07-1, defective in biosynthesis of surface polysaccharides, Kn^r^	[35]
M1116	*rmd*::Tn*5* derivative of strain XaFL07-1, defective in biosynthesis of surface polysaccharides, Kn^r^	[35]
M468	XALc_0557::Tn*5* derivative of strain XaFL07-1, defective in outer membrane protein OmpA, Kn^r^	[35]
Δ*albXXI* M8	Δ*albXXI*, defective in production of albicidin and other non-ribosomal peptide synthetase molecules	[35]
T3SS SPI-1 *xasO*	insertional mutant derivative of strain GPE PC73, defective in injectisome component of the *Salmonella* pathogenicity island-1 type 3 secretion system (SPI-1 T3SS), Kn^r^, Rif^r^	[36]
T3SS SPI-1 *xasM*	insertional mutant derivative of strain GPE PC73, defective in injectisome component of the SPI-1 T3SS, Kn^r^, Rif^r^	[36]
Δ*rpfF* M7	Δ*rpfF*, defective in production of diffusible signal factor (DSF)	[37]
Δ*rpfG* M6	Δ*rpfG*, defective in DSF receptor protein RpfG	[37]
Δ*rpfC* M29	Δ*rpfC*, defective in DSF receptor protein RpfC	[37]
Δ*rpfGCF* M15	Δ*rpfF*, Δ*rpfG*, Δ*rpfC*, defective in DSF production and receptor proteins RpfG and RpfC	[37]

### 3.2. Electroporation of *Xanthomonas albilineans* with a plasmid conferring constitutive green fluorescent protein expression

The *Kpn*I/*Sal*I fragment carrying an improved green fluorescent protein (GFP) cloning cassette [33] was excised from pUFZ75 and cloned into the same sites of pUFR047 [34] to create pUFR047-GFP (table 1). Plasmid pUFR047-GFP was then introduced into *X. albilineans* by electroporation.

Electrocompetent *X. albilineans* cells were prepared from 2-day-old liquid cultures, as described by Rott *et al*. [35]. One to 2 µl of pUFR047-GFP was electroporated into 50 µl of electrocompetent cells of *X. albilineans* in a 0.1 cm gap cuvette at 1.8 kV, 800 Ω, and a capacitance of 25 µF in a GenePulser (Biorad), with a time constant of about 9.2–9.4 ms. Following electroporation, 1 ml of liquid MW was added to the mix and the culture was grown at 28°C for 4 h at 120 r.p.m. Then, 50 µl aliquots of cultures in liquid MW were spread on MW agar plates containing gentamicin at 3 µg ml^−1^ for the selection of fluorescent colonies. *In vitro* fluorescence was confirmed by examining cells harvested from selective medium using fluorescent microscopy. Plasmid stability was assessed *in vitro* by transfer on non-selective medium followed by plating on media with and without selective antibiotics. Wild-type strains XaFL07-1 and GPE PC73 and all *Xanthomonas* mutant strains listed in table 1 were tagged with GFP using pUFR047-GFP plasmid.

### 3.3. Inoculation of sugarcane with *Xanthomonas albilineans*

Inoculation experiments were conducted at CIRAD, Montpellier, France in a biosafety level 2 (BSL-2) greenhouse using healthy plants derived from disease-free tissue-cultured plantlets of sugarcane cultivars CP68-1026, B69566, R570 and B8008, which are susceptible, moderately susceptible, tolerant and resistant to leaf scald, respectively. Plants were grown in individual 6 l pots containing a mixture of peat moss and volcanic rock (1 : 1, vol/vol). Inoculum of *X. albilineans* was prepared from 2-day-old agar cultures and bacterial suspensions were calibrated at 1 × 10^8^ CFU ml^−1^ in sterile distilled water (OD_600nm_ = 0.3–0.4 corresponding to approx. 1 × 10^9^ CFU ml^−1^). Six to 15 sugarcane stalks with at least five internodes were inoculated per strain by a modified decapitation method, as described by Rott *et al*. [35]. In this method, the spindle leaves on a stalk were cut off just below the third visible dewlap with pruning shears and 0.2–0.5 ml of inoculum was then deposited onto the cut surface.

Wild-type strains both XaFL07-1-GFP and GPE PC73-GFP were inoculated into all four sugarcane cultivars. All mutant strains of *X. albilineans* were inoculated into susceptible sugarcane cultivar CP68-1026, and sugarcane plants of this cultivar were also inoculated with non GFP-labelled wild-type strains of *X. albilineans* and were used as negative control for GFP detection and positive control for immunolocalization assays*.* Inoculation of sugarcane plants with GFP marked wild-type strains XaFL07-1 and GPE PC73 was repeated independently three times (June–August 2010, 2011 and 2012). Inoculation of sugarcane with mutant strains of *X. albilineans* XaFL07-1 (table 1) was performed once (June–August 2011).

### 3.4. Localization of bacteria *in planta* by confocal laser scanning microscopy

**Figure 1.**
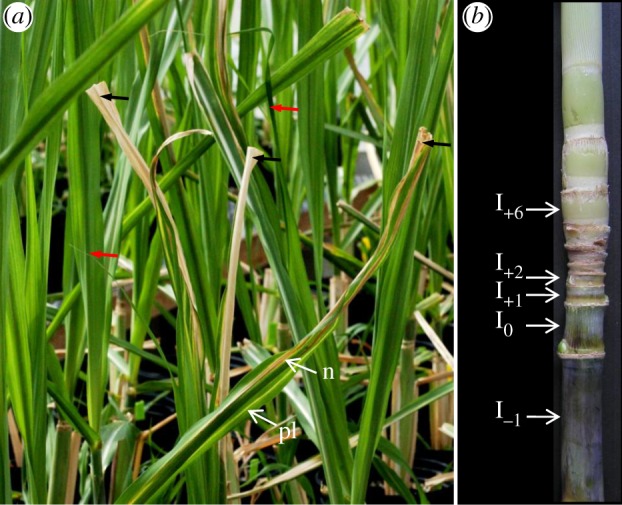
Leaf scald symptoms caused by strain XaFL07-1 of *X. albilineans* after inoculation of sugarcane plants by the modified decapitation method [35]. (*a*) Leaves with cut extremities (black arrows) are inoculated leaves, whereas leaves with uncut extremities (red arrows) are leaves that newly formed after inoculation. Inoculated leaves show pencil line streaks (pl) and necrosis (n) (photograph taken one month post-inoculation). (*b*) Infected sugarcane stalk two months post-inoculation. Note the reduced size of the internodes above internode I_0_ (i.e. first internode from the bottom with reduced size after inoculation). I, internode.

Emerging symptomatic and inoculated leaves (i.e. leaves with cut extremities) were examined 8–50 days after inoculation (figure 1*a*). These leaves were attached to nodes +2 to +5 of the stalk (figure 1*b*). At least five leaves from different sugarcane plants were examined to localize the wild-type strains of *X. albilineans* at each sampling date. Three leaves were examined for each mutant strain of the pathogen (table 1). Symptomatic leaves that newly formed after inoculation (i.e. uncut leaves that developed subsequently to inoculated cut leaves; figure 1*a*) were examined 30–60 days after inoculation. Stalk samples infected by wild-type strains XaFL07-1-GFP and GPE PC73-GFP of *X. albilineans* were examined one to six months after inoculation and were taken from above, below and at internode I_0_ (i.e. first internode from the bottom with reduced size after inoculation; figure 1*b*).

Sugarcane leaves and stalks were collected immediately prior to sectioning and sample preparation. Sections of fresh leaves (from upper and mid-third locations) and stalks (from internode location) were hand-prepared with a razor blade or obtained using a vibratome (Leica VT1000S). The leaf sections were about 60 µm thick, whereas the stalk sections were about 150–300 µm thick. Sections were then mounted on microscope slides in perfluorodecalin (C_10_F_18_, 90%; Acros Organics, Fisher Scientific) and directly examined for GFP expression with a confocal laser scanning (CLS) microscope, as described later. When sections were to be examined directly and after immunological treatment, each fresh section was divided into two parts: one half was mounted on a microscope slide in perfluorodecalin and directly examined for GFP expression with the CLS microscope, and the other half was stained with antibodies raised against *X. albilineans* [40]. For immunolocalization, all solutions were prepared in phosphate-buffered saline (PBS; Na_2_HPO_4_ 10 mM, KH_2_PO_4_ 1.7 mM, NaCl 136 mM, KCl 2.7 mM), pH 7.4. Sections of sugarcane leaves or stalks were fixed with paraformaldehyde 4% for 4 h at 4°C. After blocking in 5% bovine serum albumin solution (BSA) for 4 h at room temperature, sections were incubated in the primary antibody solution (a rabbit polyclonal antibody raised against *X. albilineans* diluted 1/800 in BSA 5%) overnight at 4°C. After washing in 0.02% Tween 20, sections were treated with the secondary antibody (an anti-rabbit Alexa594-conjugate; Promega) diluted 1/500 in 5% BSA for 1 h at 37°C. After washing with PBS, sections were mounted in perfluorodecalin and observed with the CLS microscope. Specificity of the labelling was assessed with the following controls performed on sections from healthy and infected leaves and stalks: (i) incubation with the secondary antibody only and (ii) incubation with neither primary antibody (anti-*X. albilineans*) nor secondary antibody.

Observations were carried out using a CLS microscope (Carl Zeiss LSM 700; Jena, Germany). Tissue autofluorescence, GFP and Alexa 594 were excited at 405, 488 and 555 nm, respectively. The emission signal was collected using two different tracks. Track 1 was used to visualize the GFP signal (shortpass 524 nm) and the chloroplast autofluorescence (longpass 560 nm). Track 2 was used to collect the cell wall autofluorescence (longpass 420 nm). For immunolocalization observations, fluorescence of Alexa 594 was detected using a 630 nm shortpass filter. The pictures were processed using the Carl Zeiss Confocal Software (Zen, http://microscopy.zeiss.com) and they are the maximum intensity projections of three-dimensional stacks. Characteristics of observed leaf and stalk cells are given in table 2.

**Table 2.** Characteristics of the sugarcane tissues and cells observed in this study.

plant tissue or cell type	characteristic
bulliform cells (BCs)	bubble-shaped ECs occurring in groups on the upper surface of the leaves; thought to play a role in the unfolding of developing leaves and in the rolling and unrolling of mature leaves in response to alternating wet and dry periods
epidermal cells (ECs)	cells involved in protection, support, and reduction of water loss
metaxylem (MX)	primary xylem that is last to form or develop and characterized by broader tracheary elements and web-like or pitted surfaces
non-vascular parenchyma cells (without chloroplasts) (non-VPCs)	cells involved in storage of metabolites and support
parenchyma cells with chloroplasts (also called chlorenchyma cells) or parenchyma sheath cells with chloroplasts (PSCs)	cells where photosynthesis occurs
phloem (P)	tissue with living cells which control the transport of photosynthetic products from mature leaves to other organs (notably storage organs)
protoxylem (PX)	first xylem to develop and characterized by narrow tracheary elements with annular, spiral or reticular thickenings; in young plants, the PX in stems is mostly involved in stretching or stem elongation
sclerenchyma cells (SCs)	cells involved in protection and support
storage parenchyma cells (SPCs) of the stalk	cells involved in storage of sucrose
vascular parenchyma cells (VPCs)	cells involved in storage and active transport of metabolites
xylem	tissue with vessels (dead cells with lignified cell walls), which ensure the transport of water and nutrients from the roots to the leaves

### 3.5. Localization of bacteria *in planta* by transmission electron microscopy

Leaf and stalk samples (about 2 mm^2^) were fixed for 4 h in 4% glutaraldehyde in 0.1 M cacodylate buffer pH 7.2 at 4°C, and postfixed for 1 h in 1% osmium tetroxide at 4°C in the dark. Samples were then dehydrated in a graded series of acetone and embedded in Epon resin 812 (TAAB). Ultrathin sections (60 nm) were mounted on collodion carbon-coated copper grids, contrasted using uranyl acetate and lead citrate, and examined at 80 kV with a transmission electron microscope (Jeol 100CX II).

### 3.6. Frequency of invasion by *Xanthomonas albilineans* of vascular bundles and storage parenchymatous cells in the stalk

To determine the frequency of vascular bundles and storage parenchymatous cells (SPCs) being infected by *X. albilineans*, at least three stalk sections sampled at internode –1 (upper, medium and lower third, and distant from each other by at least 1 cm) from six different stalks were examined by CLS microscopy at one month after inoculation of sugarcane cultivar CP68-1026 with strain XaFL07-1 or GPE PC73 of *X. albilineans*. The total number of vascular bundles was recorded for each section. Vascular bundles and storage parenchymatous areas invaded by fluorescent cells of *X. albilineans* were also enumerated for each section.

## 4. Results

### 4.1. *Xanthomonas albilineans* multiplies and spreads only in the xylem of sugarcane leaves during the early stages of infection

Legaz *et al*. [32] reported pathogen spread beyond xylem vessels, but this was not fully characterized. To examine this in better detail, we carried out a set of imaging experiments over both short and long time courses to examine different stages of infection. We first aimed to examine the distribution of *X. albilineans* within sugarcane leaves in the first two weeks after the inoculation of sugarcane plants with the pathogen.

**Figure 2.**
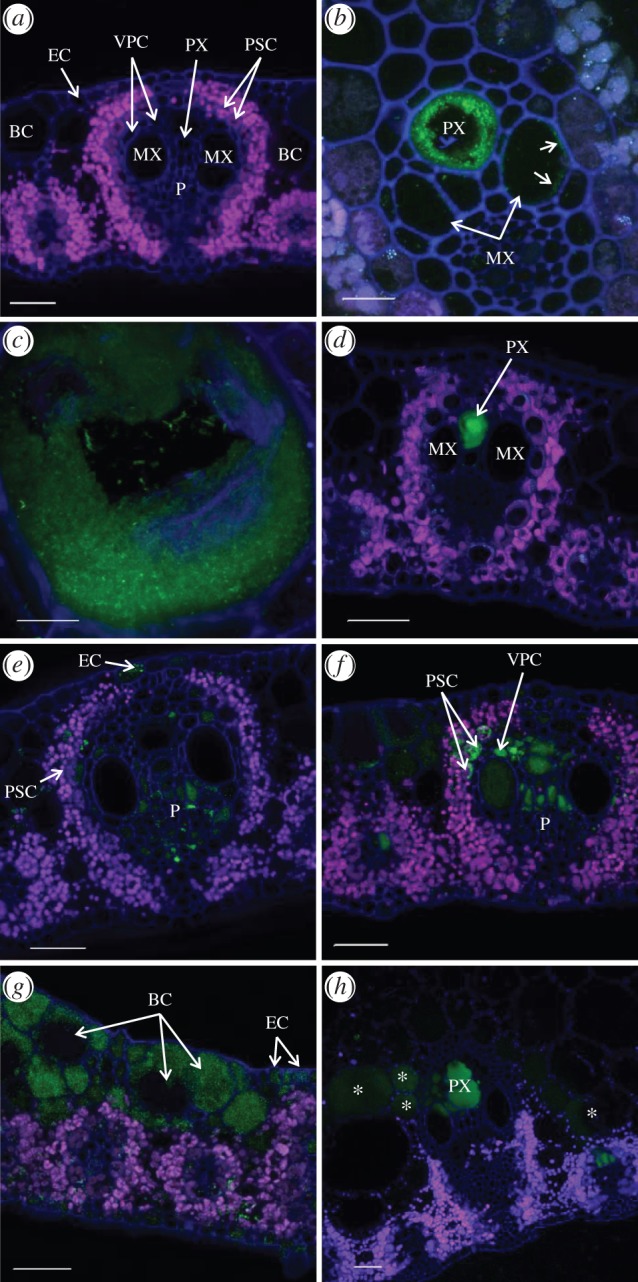
Localization of *X. albilineans* in tissues of sugarcane leaves (leaf mid-third): Confocal microscopic images of leaf cross sections of sugarcane plants inoculated with water (*a*) or *X. albilineans* (*b*–*h*). (*a*–*d*) Images were taken between 5 and 15 days post-inoculation (dpi). (*e*–*h*) Images were taken between 20 and 60 dpi. Photographs were acquired from inoculated leaves except photograph (*g*), which was acquired from a symptomatic non-inoculated leaf. (*a*) Sugarcane leaf blade inoculated with water. Vascular bundles are composed of two metaxylem vessels (MX), protoxylem (PX), phloem (P) and their associated vascular parenchyma cells (VPCs). Xylem and phloem tissues are surrounded by a ring of cells called parenchyma sheath cells with choloroplasts (PSCs). Bulliform cells (BCs) are located in the mesophyll tissue below the epidermal cells (ECs). (*b*) A thin layer of cells of the fluorescent XaFL07-1 (arrows) is seen along the inside of walls of MX cells, whereas bacteria aggregated together to form a biofilm-like structure along the walls of the PX vessel. (*c*) Thick layer of aggregated cells of the fluorescent strain XaFL07-1 of *X. albilineans* against the wall of an MX vessel. (*d*) The PX vessel is plugged with fluorescent XaFL07-1 cells. (*e*) Cells of the fluorescent strain GPE PC73 present in P cells, PSCs and ECs. (*f*) Cells of the fluorescent strain GPE PC73 present in PSCs and in VPCs. (*g*) BCs and ECs show high density of the fluorescent strain XaFL07-1. (*h*) Vascular bundles of large and small veins and the parenchyma cells surrounding these bundles (asterisks) of a sugarcane leaf midrib are invaded by cells of the fluorescent strain GPE PC73. Scale bars, 50 µm in (*a*,*d*–*h*), 20 µm in (*b*) and 10 µm in (*c*).

**Figure 3.**
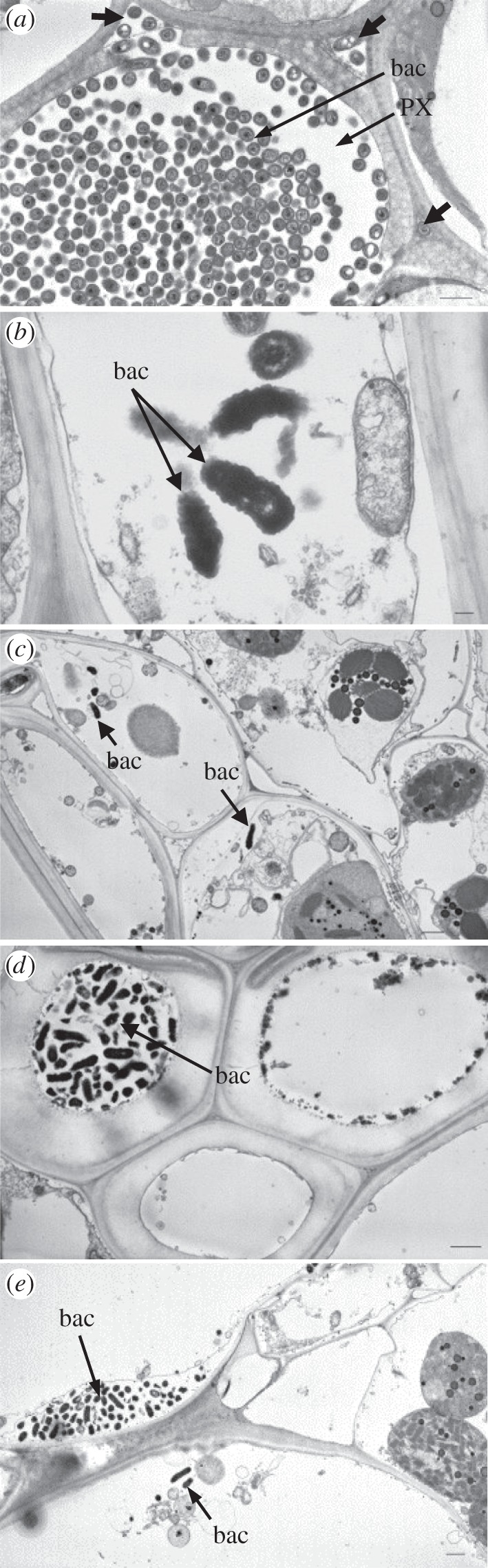
Localization of *X. albilineans* in tissues of sugarcane leaves (mid-third of inoculated leaf): TEM images of leaf cross sections of sugarcane plants inoculated with *X. albilineans* strain GPE PC73. (*a*) Lumen of a protoxylem vessel filled with cells of *X. albilineans* (bac). Note the presence of bacteria in the intercellular spaces (thick arrows) close to the vessel. Bacteria (bac) observed in (*b*) a phloem cell, (*c*) parenchyma sheath cells with choloroplasts, (*d*) sclerenchyma and (*e*) in bulliform cells. Note the presence of bacteria (bac) in the periplasmic space in the upper left cell (*e*). Scale bars, 1 µm in (*a,c*–*e*) and 100 nm in (*b*).

Five to 15 days post-inoculation (dpi), wild-type strains XaFL07-1 and GPE PC73 of *X. albilineans* constitutively expressing a GFP were observed by confocal laser scanning microscopy (CLSM) in xylem vessels (protoxylem (PX) and metaxylem (MX); table 2 and figure 2*a*) of sugarcane leaves of the susceptible cv. CP68-1026 (figure 2*b*). Accurate examination of these infected vessels revealed occurrence of three major and different distributions of the pathogen: (i) aggregated bacterial cells that formed a thin layer of a biofilm-like structure along the PX and/or MX wall (figure 2*b*), (ii) large aggregates of bacteria that formed a thick layer along the xylem vessels, although individual bacteria still remained in the xylem lumen (figure 2*b,c*) and (iii) bacteria that were packed together and filled xylem vessels (figures 2*d* and 3*a*). At this stage of leaf invasion, the fluorescent bacteria were only seen in the xylem.

### 4.2. *Xanthomonas albilineans* multiplies and spreads in the phloem, the vascular parenchyma cells and various cells of non-vascular tissue during disease progress in the leaves

When leaves were examined at later stages of infection (i.e. 20–60 dpi), wild-type strains XaFL07-1 and GPE PC73 of *X. albilineans* were found in tissues other than the xylem (table 2). Bacteria were then observed in phloem cells (figures 2*e* and 3*b*), in parenchyma sheath cells with chloroplasts (PSCs; figures 2*f* and 3*c*), in vascular parenchyma cells (VPCs; figure 2*f*), in sclerenchyma (figure 3*d*) and in bulliform cells (BCs) and ECs (figures 2*g* and 3e). Bacteria were also observed in the vascular bundles and in non-VPCs in the leaf midrib (figure 2*h*). When bacteria were seen in the phloem, the parenchyma sheath or in BCs, adjacent xylem vessels were not systematically infected (figure 2*e*). Like in PX and MX vessels, high densities of bacteria sometimes occurred in the phloem, in VPCs and in non-vascular cells.

**Figure 4.**
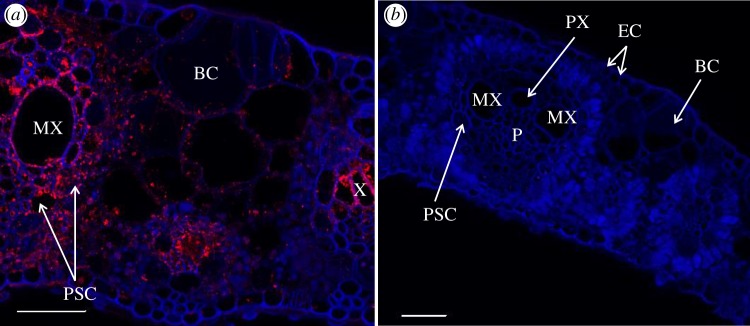
Immunochemistry confirming localization of *X. albilineans* in tissues of sugarcane leaves (mid-third of inoculated leaf): confocal microscope images of transverse leaf blade sections of sugarcane plants inoculated with (*a*) *X. albilineans* strain GPE PC73 and (*b*) water (taken at 30 dpi). The sections treated with antibodies raised against *X. albilineans* reveal red-stained bacteria. MX, metaxylem; PX, protoxylem; P, phloem; PSCs, parenchyma sheath cells with choloroplasts; BC, bulliform cell; EC, epidermal cell. Scale bars, 50 µm in (*a,b*).

The same pattern of tissue colonization was also observed in the moderately susceptible B69566 and tolerant R570 sugarcane cultivars after inoculation with the two wild-type strains of the pathogen (XaFL07-1 and GPE PC73) labelled with GFP. Additionally, the presence of *X. albilineans* outside of the xylem was also confirmed by immunolocalization (figure 4*a*). Furthermore, these different localizations of the pathogen in sugarcane leaf tissues were seen in symptomatic inoculated leaves and in symptomatic non-inoculated leaves that became infected after systemic infection of the sugarcane stalk by the pathogen. No fluorescent bacterial cells (GFP signal or after immunolocalization) were observed in control leaves inoculated with water (figures 2*a* and 4*b*).

### 4.3. Pathogenicity mutants of *Xanthomonas albilineans* also multiply and spread in the xylem and in other tissues of sugarcane leaves

To understand the role of some candidate pathogenicity genes during invasion of sugarcane by *X. albilineans*, GFP-labelled mutant strains of *X. albilineans* (table 1) were localized by CLSM in sugarcane leaves of cv. CP68-1026 at 30–60 dpi. Similar to the wild-type strains of *X. albilineans*, the mutant strains affected in surface polysaccharide production, non-ribosomal peptide synthetases production, diffusible signal factor (DSF) production, the DSF sensor/regulator system, the *Salmonella* pathogenicity island-1 (SPI-1) type 3 secretion system and in outer membrane protein A synthesis were all able to invade xylem vessels, phloem, parenchyma and BCs. However, polysaccharide mutants (four strains) and the XaOmpA1 mutant were found only in leaf sections that were sampled near the cut leaf surface (top of upper third leaf) that is considered to be the entry point of the pathogen after decapitation of the sugarcane stalk and inoculation of sugarcane leaves. These latter mutants invaded only a few vascular vessels in comparison with the wild-type strains of *X. albilineans* (data not shown). This result was not surprising as these mutants are affected in the production of symptoms and in their capacity to spread in the sugarcane stalk [35,41].

### 4.4. *Xanthomonas albilineans* multiplies in the xylem, the surrounding vascular parenchymatous cells and in the phloem of the stalk

Martin *et al*. [30] reported pathogen spread beyond xylem vessels in the stalk and formation of lysigenic cavities, but this was not fully characterized. To examine this in better detail, we carried out a set of imaging experiments over both short and long time courses to examine different stages of stalk infection.

**Figure 5.**
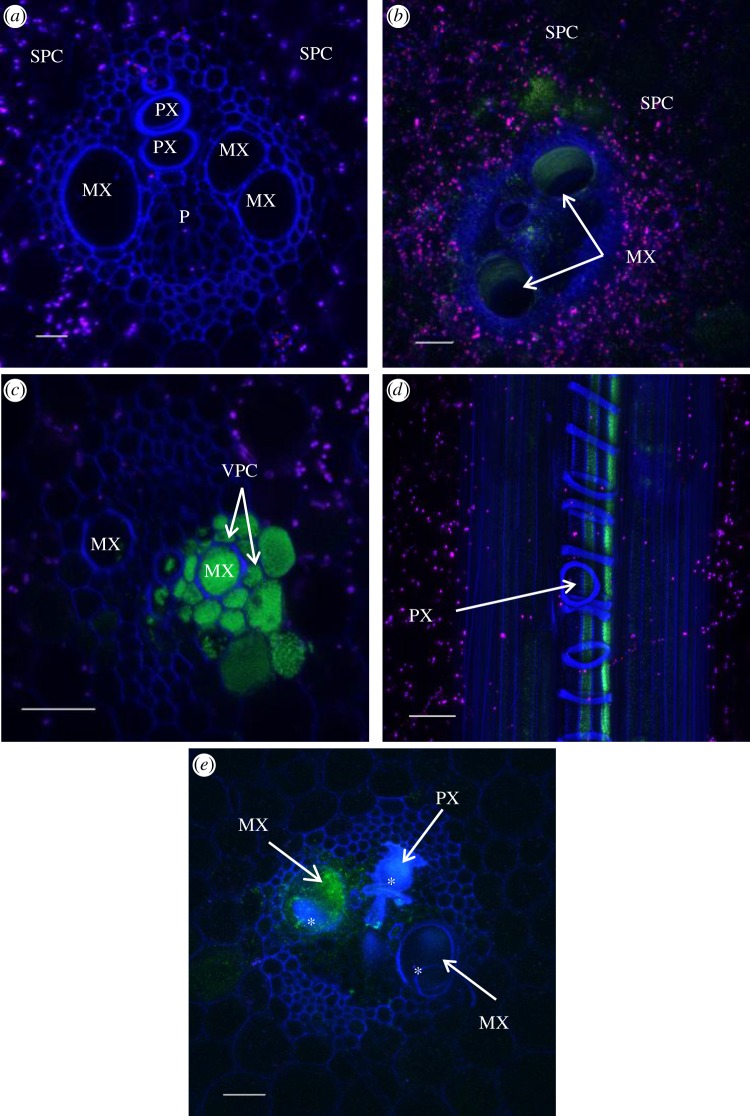
Localization of *X. albilineans* in vascular tissues of sugarcane stalks: confocal microscopic images of internode cross sections of sugarcane plants inoculated with *X. albilineans.* Photographs were taken between two and four months post-inoculation (mpi). (*a*) Cross section of sugarcane stalk inoculated with water showing a vascular bundle and associated storage parenchymatous cells. MX, metaxylem; PX, protoxylem; P, phloem; SPCs, storage parenchymatous cells (stalk internode –1). (*b*) Cells of the fluorescent strain XaFL07-1 are organized as a thin layer of a biofilm-like structure along MX vessels (internode 0). (*c*) Strong green fluorescence in the vascular parenchyma cells (VPCs) surrounding xylem vessels (MX) indicating the presence of bacterial cells of strain XaFL07-1 (internode –6). (*d*) Longitudinal section showing a stream of the fluorescent strain XaFL07-1 cells along a PX vessel (internode 0). (*e*) PX and MX vessels partially or completely plugged with a blue-stained matrix (asterisks) in which the fluorescent bacteria (strain GPE PC73) are embedded (internode 0). Scale bars, 20 µm in (*a*), 50 µm in (*b*–*d*) and 100 µm in (*e*).

**Figure 6.**
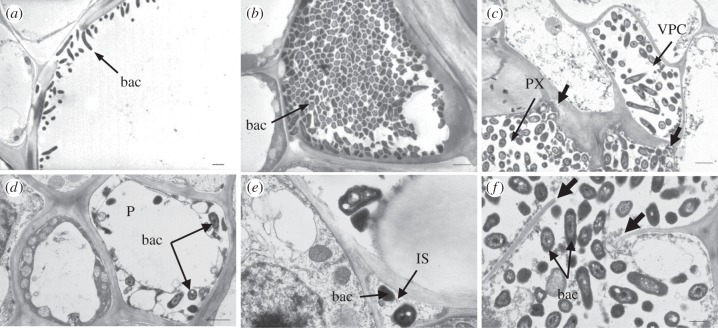
Localization of *X. albilineans* in tissues of sugarcane stalks: TEM images of internode cross sections of sugarcane plants inoculated with *X. albilineans.* (*a,b*) was taken between two and four months post-inoculation (mpi) and (*c*–*f*) was taken at 5 mpi. (*a*) Thin layer of rod-shaped bacterial cells (bac) of strain GPE PC73 along a metaxylem vessel (stalk internode –1). (*b*) Densely packed bacteria (strain GPE PC73) in a xylem vessel; bacteria (bac) are no longer rod-shaped but show a polygonal structure (internode –1). (*c*) Presence of the bacteria (strain Δ*rpfC* M29) in the vascular parenchyma cells (VPCs) surrounding protoxylem (PX) vessels (internode +24). Thick arrows in (*c*) indicate plant cell wall lysis. (*d*) A phloem cell (P) filled with bacterial cells (bac) (strain Δ*rpfC* M29) (internode +24). (*e*) Occurrence of *X. albilineans* (bac) (strain Δ*rpfC* M29) in intercellular spaces (IS) between VPCs (internode +24). (*f*) Rupture (thick arrows) in the cell wall of a VPC filled with bacterial cells (strain Δ*rpfC* M29) (bac) (internode +24). Scale bars, 1 µm in (*a*–*d*) and 0.5 µm in (*e,f*).

**Figure 7.**
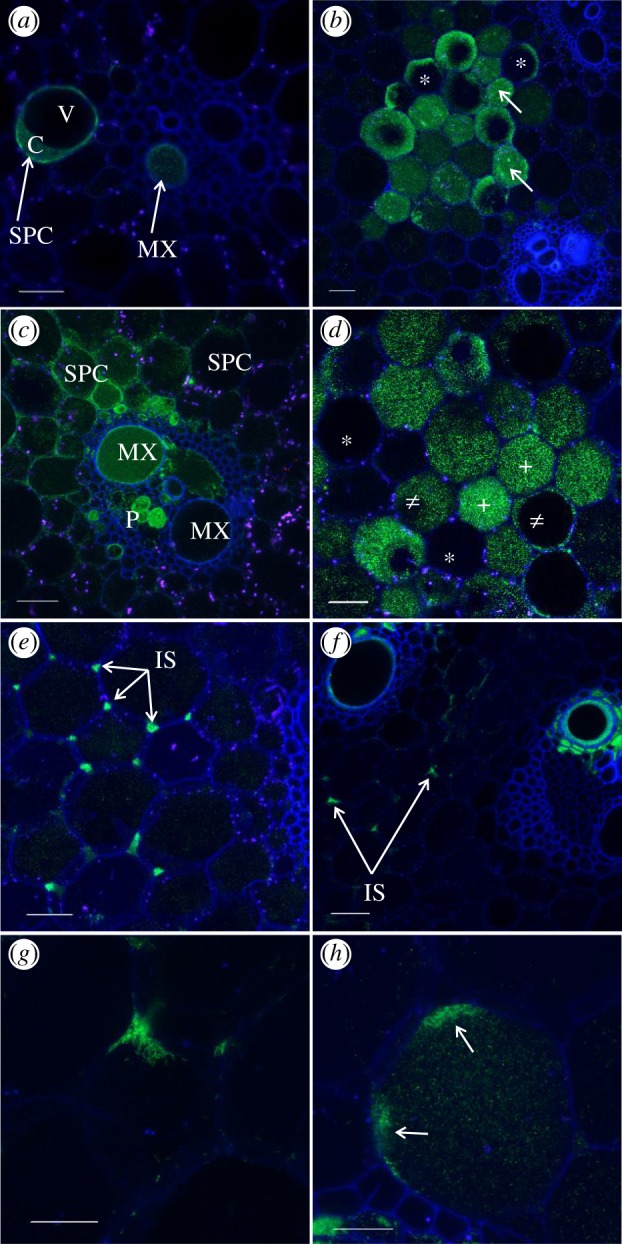
Occurrence of *X. albilineans* in storage tissues of sugarcane stalks: confocal microscope images of internode cross sections of sugarcane plants inoculated with *X. albilineans**.*** Photographs were taken between two and four months post-inoculation. (*a*) Occurrence of fluorescent strain XaFL07-1 in a metaxylem (MX) vessel and outside the vascular bundle in a storage parenchyma cell (SPC) (stalk internode –4). The bacteria occupy the cytoplasm (C) of the SPC but the vacuole (V) appears free of bacteria. (*b*) SPCs that are filled with the fluorescent XaFL07-1 cells (arrows), whereas others appear only weakly colonized (asterisks) (internode –1). (*c*) Vascular bundles (MX and phloem (P)) and surrounding SPCs colonized by cells of the fluorescent strain GPE PC73 (internode –1). (*d*) SPCs that are apparently free of the pathogen (asterisks), partially (#) or completely filled (+) with cells of the fluorescent GPE PC73 (internode –2). (*e*) The strong green fluorescence in intercellular spaces (IS) of SPCs reveals the presence of bacterial cells of strain GPE PC73 (internode –2). (*f*–*g*) The fluorescent strain XaFL07-1 located in the IS and along the cell wall facing those spaces (arrow). Note that these cells do not seem to be colonized (*f,g*, internode +11). (*h*) An SPC invaded by the fluorescent bacteria (strain XaFL07-1). High population densities are observed along the cell wall facing IS (arrows) (internode 0). Scale bars, 50 µm in (*a,c*–*f*), 100 µm in (*b*) and 20 µm in (*g,h*).

**Figure 8.**
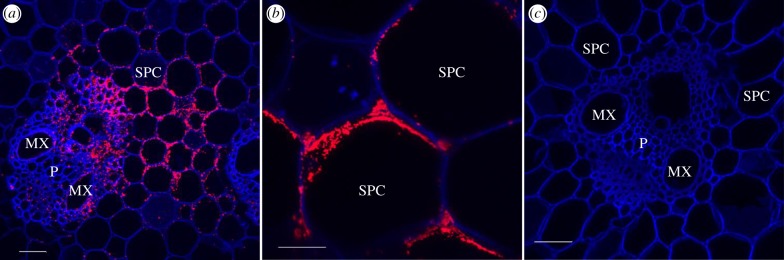
Immunochemistry confirming the localization of *X. albilineans* in storage tissues of sugarcane stalks: confocal microscope images of transverse sections of stalk internodes of sugarcane plants inoculated with *X. albilineans* strain GPE PC73 (*a,b*) (stalk internode –1) and water (*c*) (internode –2); photographs were taken at two months post-inoculation. The sections (*a*–*c*) treated with antibodies raised against *X. albilineans* display red-stained areas revealing the presence of the bacteria in infected tissues. MX, metaxylem; P, phloem; SPC, storage parenchyma cell. Scale bars, 50 µm in (*a,c*) and 20 µm in (*b*).

One and two months post-inoculation (mpi), the wild-type strains XaFL07-1-GFP and GPE PC73-GFP of *X. albilineans* and the mutant Δ*rpfC* M29-GFP were seen in xylem vessels (PX and MX) using CLSM, but also in storage parenchymatous tissue of sugarcane stalks of cv. CP68–1026 (figures 5–8). At 5 mpi, bacteria were found in different internodes from I_–6_ (i.e. 6th internode below I_0_, the first internode with reduced size that grew after inoculation; figure 1*b*), to the internode just below the apical meristem (up to I_+24_).

Different patterns of tissue invasion were found in the sugarcane stalk that suggested the following infection process. At initial vascular invasion of the stalk through colonized inoculated leaves, bacterial cells grow and aggregate to form a thin layer of a biofilm-like structure that adheres to the MX and/or PX wall (figure 5*b*). At this invasion stage, individual and rod-shaped bacteria that are similar to their free-living form were also seen in the xylem by TEM (figure 6*a*). After additional growth within the xylem, bacterial populations became very dense and colonized vessels were filled with bacteria (figures 5*c* and 6*b*). A stream of bacterial cells along the xylem vessel was observed by CLSM in longitudinally sectioned samples (figure 5*d*). At this stage, bacteria were densely packed together and their structure was different when compared with free-living cells. In plugged vessels, transversally sectioned bacteria showed by TEM a polygonal structure (figure 6*b*), whereas initial free-living bacteria were rod-shaped (figure 6*a*). Xylem vessels were also frequently partially or completely occluded with a matrix, in which bacteria were sometimes seen localized (figure 5*e*). However, these bacteria were often pale green by CLSM, suggesting that these bacteria were almost dead or metabolically inactive.

As observed by TEM, VPCs surrounding the invaded xylem vessels generally conserved their integrity and organelles. However, in some cases, bacteria were viewed in these cells (figure 5*c*), which were then highly disorganized (no distinguishable organelles) and distorted (loss of turgescence) (figure 6*c*). Bacteria were also sometimes observed in phloem cells (figure 6*d*). Infected phloem cells were not heavily altered and organelles were still distinguishable in these cells. However, when compared with the extent of xylem tissue colonization, the phloem elements were rarely seen invaded by the pathogen. Bacteria also occurred in the intercellular spaces between the xylem and the VPCs (figure 6*e*). Vascular bundles neighbouring infected ones were often not colonized by the bacteria and their morphology and organization appeared similar to healthy vascular bundles.

### 4.5. *Xanthomonas albilineans* multiplies in the storage tissue of sugarcane stalks

Cells of *X. albilineans* were also found by CLSM outside the vascular bundles and bacteria were seen inside SPCs, which are located between the vascular bundles of the stalk. Bacteria initially occupied the cytoplasm of these cells (figure 7*a*) and then invaded the entire area of the cell, most likely by dissolving the tonoplast (figure 7*b–d*). Bacteria were also found within the intercellular spaces of the storage parenchyma where populations were sometimes very dense, as highlighted by a strong GFP signal (figure 7*e,f*). In parenchymatous cells starting to be invaded by the pathogen, bacterial cells were only observed along the cell wall facing intercellular spaces, suggesting that the pathogen enters the plant cell at these sites (figure 7*g***,***h*). Infected storage cells were frequently located in the vicinity of invaded vascular bundles but not always, and the bacteria were also observed in storage cells neighbouring apparently bacteria-free xylem. However, although invaded parenchyma cells were not always seen adjacent to an invaded vascular bundle, this proximity may exist somewhere else around the whole cell or existed at an earlier stage of tissue development. The occurrence of cells of *X. albilineans* in SPCs was confirmed by immunolocalization (figure 8*a,b*).

During the late stage of the infection (4–6 mpi), TEM observations of stalk sections sampled just below the apical meristem suggested extensive bacterial multiplication, which was often associated with a fibrillar matrix and ultrastructural destruction of xylem elements. In VPCs, organelles were highly altered and thus no longer distinguishable. Primary walls were initially degraded (figure 6*c*) and then locally completely fragmented, which enabled the bacteria to spread within the adjacent vessels or cells (figure 6*f*). Cell or vessel structure disappeared partially or completely and, as a consequence, xylem vessels and parenchyma cells could not be differentiated. These areas became then filled with bacteria and fragments of the cell walls and organelles. In these highly disintegrated areas, phloem cells appeared intact and only few showed signs of degradation.

The same pattern of stalk colonization was also observed in sugarcane cultivars B69566, R570 and B8008 after inoculation with the two wild-type strains of the pathogen (XaFL07-1 and GPE PC73) labelled with GFP. However, the number of colonized vascular bundles of the resistant cultivar B8008 (no more than 1% of bundles) was lower than that in the three other cultivars (more than 10% of bundles). No fluorescent bacterial cells (GFP signal or after immunolocalization) were observed in control stalks inoculated with water (figures 5*a* and 8*c*).

Frequency of stalk invasion by *X. albilineans* was investigated at internode –1, one month after the inoculation of cultivar CP68-1026. Each stalk section contained a mean of 470 vascular bundles (18 sections observed), among which 28 were infected by *X. albilineans*. A mean of seven storage parenchymatous areas per section were also found infected by fluorescent bacteria, which corresponds to one infected parenchymatous location for every four infected vascular bundles.

## 5. Discussion

Based on microscopy observations using three cytological approaches, we show for the first time that a plant vascular bacterium with reduced genome, supposed to be strictly localized in vessels of the infected host, was also detected in other leaf and stalk tissues. Confocal microscopy, immunocytochemistry and TEM revealed the occurrence of the sugarcane pathogen *X. albilineans* in xylem elements, phloem, sclerenchyma, epidermal and various parenchymatous cells.

This finding that *X. albilineans* is able, after invading xylem vessels and multiplying in intercellular areas, to penetrate into apparently intact vascular parenchymatous cells and other non-vascular plant tissues contradicts the current knowledge regarding the habitat of plant pathogenic bacteria. In contrast to mammalian bacterial pathogens which can invade their host cells [42], cultivable plant pathogenic bacteria are known to spend most of their parasitic life in xylem elements (vascular pathogens) or in the intercellular areas of the mesophyll tissue (apoplastic pathogens) [43]. Invasive strategy of intracellular mammalian bacterial pathogens, which replicate within spacious phagosomes in macrophages, relies on effector proteins delivered by T3SS that allow bacterial uptake into host cells, enhance intracellular replication and remodel the vacuole into a replicative niche [42]. Bacterial plant pathogens have also evolved specific strategies to establish themselves successfully in their hosts and to acquire nutrients from the plant cells [2,43,44]. Most Gram-negative plant pathogenic bacteria deploy an Hrp T3SS to inject effector proteins directly into the plant cell cytosol to interact with various intracellular targets and modulate host cell processes from outside the plant cells [45]. The few bacterial plant pathogens that are missing this T3SS have a limited niche in the host plant. It has been proposed that *L. xyli* subsp. *xyli* was once a free-living bacterium that is now restricted to the xylem as a consequence or cause of the loss of functions associated with pseudo-genes [13]*.* Similarly, Simpson *et al*. [15] suggested that the T3SS is not required by *Xylella fastidiosa* because of the insect-mediated transmission and the xylem restriction of the bacterium that obviates the necessity of host cell infection. The same explanation was given for phytoplasma, which are phloem-restricted microorganisms directly introduced into the phloem cells by their insect vectors [24].

As the sugarcane pathogen *X. albilineans* is missing the Hrp T3SS and also the genes involved in the biosynthesis of xanthan gum [7], a key pathogenicity factor that protects intercellular *Xanthomonas* species from environmental stresses [46,47], our results raise a main question: which are the underlying mechanisms that allow *X. albilineans* to invade numerous and diverse host cell types, especially non-vascular tissues? Pieretti *et al*. [8] proposed, on the basis of comparative genomic analyses, that *X. albilineans* most likely uses specific strategies to avoid detection or to protect itself against sugarcane defence mechanisms. This hypothesis is supported by a study showing that the plant nitrogen-fixing endophytic bacterium *Gluconacetobacter diazotrophicus* produces elicitor molecules that activate the sugarcane defence responses resulting in plant resistance to *X. albilineans* [48].

The mechanisms used by *X. albilineans* to exit the xylem and to invade the parenchyma cells remain to be unravelled. Pathogenicity factors, such as albicidin toxin, quorum sensing DSF, outer membrane protein A, SPI-1 T3SS and surface polysaccharides, do not appear to play a direct role in this phenomenon. To exit xylem vessels, *X. albilineans* may cause the rupture of the cell wall by chemical dissolution of vessel primary and secondary walls, and the middle lamellae. Host cell wall alteration observed by TEM in the advanced stage of sugarcane infection reinforces the idea that the bacteria secrete degrading enzymes that will soften the cell wall thus facilitating the progression of the pathogen towards the adjacent cells. Plant pathogenic bacteria, including species of the genera *Erwinia* [49,50], *Clavibacter* [51] and *Xanthomonas* [52], were previously described to possess an enzymatic arsenal most probably associated with pathogenesis and plant cell wall degradation. The genome of *X. albilineans* possesses 19 genes encoding putative cell wall degrading enzymes [8].

Further studies are needed to investigate the role of these enzymes in intracellular invasion of sugarcane cells by *X. albilineans*. Similar studies of pathogenicity factors of other bacterial plant pathogens that experienced genome reduction, such as *X. fragariae* [53] or pathogenic *Xanthomonas* species isolated from banana that are missing an Hrp T3SS [54,55], may also contribute to understand the invasion strategy of plant bacteria that are so far considered as strict vascular pathogens.

## Supplementary Material

Imprints of sugarcane stalk sections on selective medium
