# Supplementary material for: Breaking dogmas: the plant vascular pathogen Xanthomonas albilineans is able to invade non-vascular tissues despite its reduced genome
Source: Open Biol. 2014 Feb 12;4(2):130116. doi: 10.1098/rsob.130116 (PMC3938051; doi:10.1098/rsob.130116)
Supplement: Imprints of sugarcane stalk sections on selective medium [file rsob130116supp1.pdf]

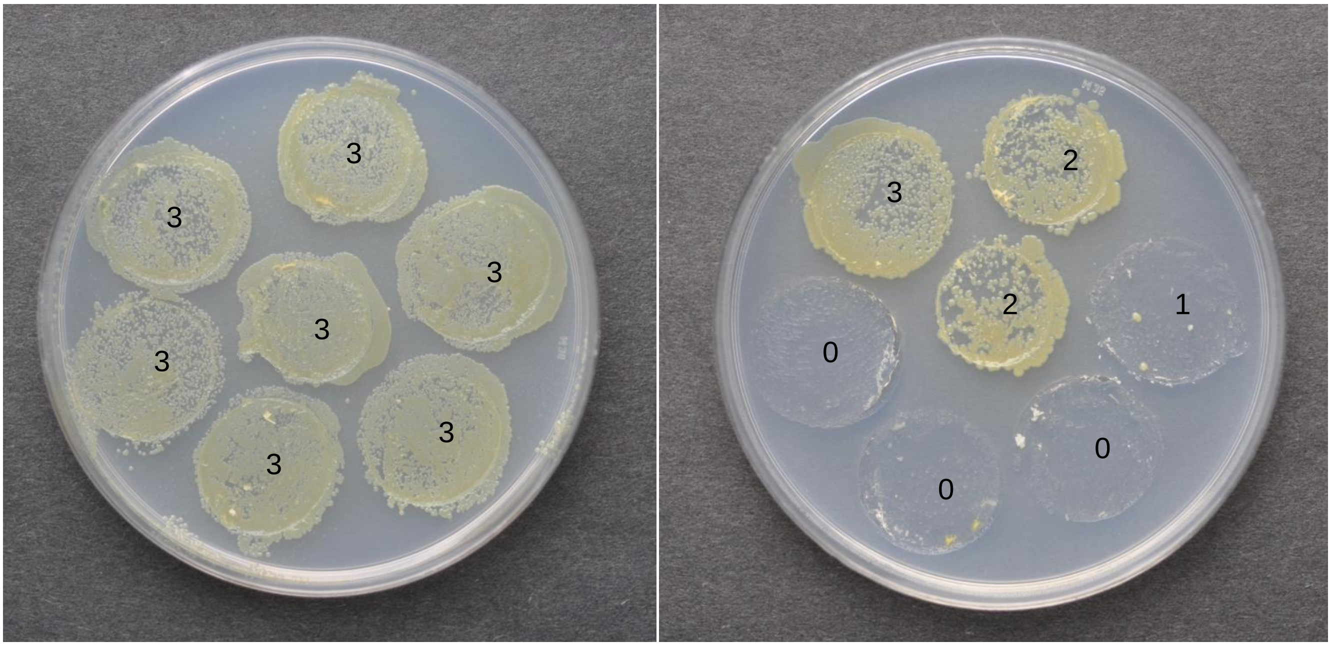

Electronic Supplementary Material (ESM)

Figure 1. Imprints of sugarcane internodes inoculated by *Xanthomonas albilineans* strain XaFL07-1: Growth of *Xanthomonas albilineans* strain XaFL07-1 after inoculation of selective medium by the stalk blot inoculation technique two months post inoculation [34]. Surface sterilized sugarcane stalks were cut transversally, in the middle of internodes, with sterile pruning shears. Each cut sections was firmly pressed onto WCNCB medium (= MW agar medium supplemented with 25 mg/l cephalexin, 30 mg/l novobiocin, 50 mg/l cycloheximide, and 12.5 mg/l benomyl). Growth of *X. albilineans* was recorded after 5 days of incubation of agar plates at 30°C. Growth patterns of the bacteria reveal different distributions and population densities of the pathogen in the stalk: 0 = no bacterial colony in the stalk imprint (absence of the pathogen), 1 = growth of few single colonies in the stalk imprint indicating colonization of few vascular bundles, 2 = growth of numerous single colonies in the stalk imprint suggesting colonization of numerous vascular bundles, 3 = confluent growth of bacteria suggesting colonization of numerous vascular bundles and occurrence of the bacteria outside of the xylem.
